# Binding of interferon reduces the force of unfolding for interferon receptor 1

**DOI:** 10.1371/journal.pone.0175413

**Published:** 2017-04-12

**Authors:** Silvia G. Chuartzman, Reinat Nevo, Sharon Waichman, Dalit Shental, Jacob Piehler, Yaakov Levy, Ziv Reich, Ruti Kapon

**Affiliations:** 1 Department of Biomolecular Sciences, Weizmann Institute of Science, Rehovot, Israel; 2 Department of Biology, University of Osnabrück, Osnabrück, Germany; 3 Department of Structural Biology, Weizmann Institute of Science, Rehovot, Israel; Russian Academy of Medical Sciences, RUSSIAN FEDERATION

## Abstract

Differential signaling of the type I interferon receptor (IFNAR) has been correlated with the ability of its subunit, IFNAR1, to differentially recognize a large spectrum of different ligands, which involves intricate conformational re-arrangements of multiple interacting domains. To shed light onto the structural determinants governing ligand recognition, we compared the force-induced unfolding of the IFNAR1 ectodomain when bound to interferon and when free, using the atomic force microscope and steered molecular dynamics simulations. Unexpectedly, we find that IFNAR1 is easier to mechanically unfold when bound to interferon than when free. Analysis of the structures indicated that the origin of the reduction in unfolding forces is a conformational change in IFNAR1 induced by ligand binding.

## Introduction

Signal activation by cytokine receptors is initiated by interaction of a ligand with two or more receptor subunits. However, the mechanism of signal propagation across the membrane remains debated: while initially ligand-induced receptor dimerization was suggested to initiate signaling, a refined picture which includes a subtle interplay of interaction and conformational changes with, possibly, pre-dimerization of the receptor, is emerging [1, 2]. The mechanism of signal activation is inextricably linked to the ability of cytokine receptors to be differentially activated by different ligands. The type I interferon (IFN) receptor (IFNAR) is a paradigm for such functional plasticity of cytokine receptors, as a large family of IFNs (17 members in humans) bind to the same cell surface receptor yet elicit non-redundant spectra of anti-viral, anti-proliferative and immunomodulatory responses [3–5]. IFNAR is comprised of two subunits, IFNAR1 and IFNAR2 that interact with a single IFN molecule to form the ternary signaling complex. While IFNAR2 binds all IFNs with high affinity (lower nanomolar *K*_*D*_) and therefore is considered to be responsible for ligand binding to the cell surface, IFNAR1 recognizes IFNs with approximately three orders of magnitude lower affinity (micromolar *K*_*D*_), which substantially varies for different members of the family. Detailed structure function analyses have identified the interaction of IFN with IFNAR1 as a critical determinant for IFN receptor plasticity [6–9]. This can be ascribed to the low affinity interaction of IFNs with IFNAR1 being the limiting factor for ternary complex formation in the plasma membrane [10]. In addition, ternary complex formation at the plasma membrane is modulated by the negative feedback regulator USP18 [10, 11], which has been shown to play a decisive role in functional plasticity of IFNAR [11, 12]. These results suggest that recognition of IFNs by IFNAR1 has evolved to provide a broad spectrum of binding properties with respect to stability and/or conformational dynamics of ternary complex to fine-tune cellular responses upon infection by different pathogens [13–15].

**Fig 1:**
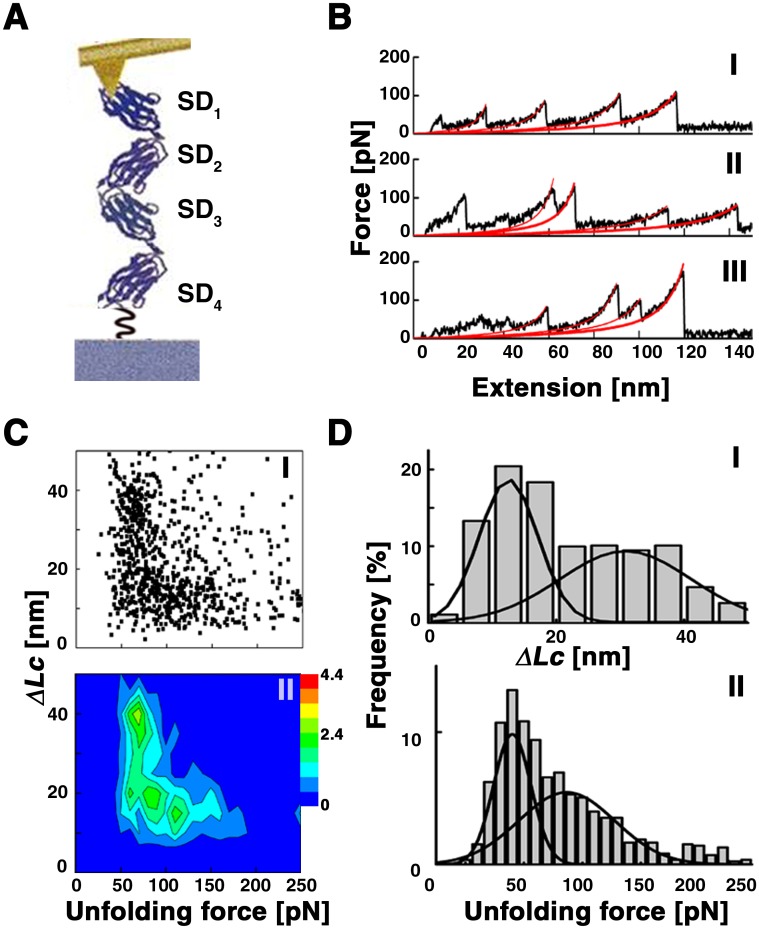
Forced unfolding of IFNAR1-EC by AFM. (A) The C-terminal of IFNAR1-EC immobilized onto a mica surface through a flexible linker and an AFM tip interacting with the protein (B) Traces representative of different families of unfolding curves. (C) I. A scatter plot representing the phase space of the system in the *ΔL*_*C*_ vs. *F* plane. II. Corresponding contour plot (D) I. Histogram of contour length changes fitted by two Gaussians centred at 16 nm and 36 nm with a p-value < 10^−4^. II. The force histogram corresponding to the events in I fitted with Gaussians centred at 40 pN and 90 pN.

The extracellular domain of IFNAR1 is unique among the class-II cytokine receptors in that it contains four fibronectin type-III (FNIII)–like domains in tandem [16–18] (SD1-SD4) (see Fig 1). All domains have the characteristic seven β strands that are arranged in a β-sandwich of two sheets. The N-terminal pair of domains, SD1-SD2, bears a structural similarity to SD3-SD4 with a comparable positioning of the cysteine disulfide bond present in each domain. Of the four domains, the three N-terminal ones (SD1-SD3) are involved in IFN binding [9, 19–21]. The fourth, membrane proximal domain, SD4, is thought to be required for assembly of the ternary complex on the membrane [19] and has been suggested to interact with IFNAR2 [20]. Upon IFN binding, IFNAR1 undergoes a conformational change that involves movement of SD1 relative to SD2-SD3, effectively capping the ligand [9, 22] (bound and unbound structures can be viewed in proteopedia, http://proteopedia.org/wiki/index.php/IFNAR). Interestingly, this conformational change is propagated to the membrane proximal domain SD4 [22], highlighting its possible role in communication across the membrane. In line with the occurrence of complex conformational changes upon ligand binding, temperature-dependent binding studies revealed an apparent negative activation energy the IFN-IFNAR1 interaction [22]. To further shed light into the structural determinants controlling IFN recognition by IFNAR1, we explored the forced unfolding of IFNAR1 on its own and when bound to IFN, using an atomic force microscope (AFM) and molecular dynamics (MD) simulations.

## Materials and methods

### Protein biochemistry

IFNAR1-EC with a C-terminal decahistidine tag (IFNAR1-H10) was expressed in Sf9 insect cells (Gibco), and purified from the supernatant by immobilized metal ion affinity chromatography followed by size exclusion chromatography as described earlier [8].

Site-directed mutagenesis of IFNα2 was carried out by primer extension using bacteriophage T7 DNA polymerase on expression vector pT7T318U containing the IFNα2 gene, which was used as a single-stranded DNA template following subsequent transfection into and recombinant protein production in CJ236 cells [23, 24].

IFNα2 and IFNα2 NLYY were expressed in *E*. *coli* BL21 Rosetta (Novagen), refolded from inclusion bodies and purified by anion–exchange and size-exclusion chromatography as described [23, 25]. IFNα2 YNS was also expressed in the *E*. *coli* BL21 Rosetta, but was refolded by an alternative protocol: following four washing cycles with Triton wash solution (0.5% Triton X-100, 50 mM Tris, pH 8.0, and 100 mM NaCl) and one additional wash without Triton, inclusion bodies were solubilized in 6 M guanidine hydrochloride and then refolded by 1:20 dilution in 0.8 M arginine solution, pH 9.3, followed by dialysis against 25 mM Tris, pH 7.4. The protein was then purified by ion-exchange chromatography followed by size exclusion chromatography [8].

Structural and functional integrity of IFNAR1-EC was confirmed by analytical size exclusion chromatography in presence and in absence of the high affinity IFNα2 YNS mutant (S1 Fig).

All IFN constructs have the same isoelectric point.

### Surface modification

H10-tagged IFNAR1-EC (IFNAR1-H10) was immobilized to a mica surface through a flexible polymer linker which allows the protein to explore different directions relative to the mica surface. The linker was functionalized with tris-nitrilotriacetic acid (tris-NTA) [26] for rapid and stable site-specific tethering of IFNAR1-H10 via the H10-tag.

Freshly cleaved V1 grade mica (Ted Pella) was reacted with ethanolamine- HCl (Sigma–Aldrich), dried and incubated for 2 h with 6mg/ml of ~8.3 Å long SMCC(*N*-Succinimidyl 4-(maleimidomethyl)cyclohexanecarboxylate) in chloroform containing 0.5% of triethylamine (Sigma-Aldrich). After three washes with chloroform, the dried mica was incubated with 2–3 nm long HS-PEG7-Tris NTA, which was prepared from the reduction of the corresponding disulfide [27] (500 μM) with 2 mM tris(2-carboxyethyl)phosphine (TCEP) in HBS buffer (20 mM Hepes pH = 7.5, and 150 mM NaCl). The mica was then washed with DDW, dried and kept at -20°c until use. Immediately prior to the experiment, the tris-NTA was loaded with nickel (II) (10 mM NiCl_2_ in HBS buffer) followed by incubation with IFNAR-H10 in HBS buffer with 0.01% Triton-X100 for 30 min [28]. The latter was added to avoid unspecific interactions of the protein with the mica. Finally, the sample was washed in HBS buffer with 0.01% Triton-X100 to remove unbound material. IFNα2 WT and mutants were all added to a final concentration of 4 μM.

### Mechanical unfolding using the AFM

Measurements were carried out at room temperature (22–25°C) in HBS buffer with 0.01% Triton X-100, on a PicoSPM AFM (Molecular Imaging, Agilent Technologies), equipped with a liquid cell, using silicon nitride cantilevers (MSCT-AUHW, Veeco Instruments). The spring constants of the cantilevers were determined by measuring the amplitude of their thermal fluctuations [29], and were in the range of 0.018–0.038 N/m. Most unfolding experiments were conducted at a pulling speed of 200 nm/sec (but see force spectra in S3 Fig for forces measured when pulling at other speeds). The first peak, which many times reflects tip-adhesion to the surface, and the last peak which reflects detachment of the protein from the tip or from the surface were excluded from the analysis. Analysis was performed on 200–400 traces obtained from three to four independent experiments which exhibited at least two clear sequential unfolding events. The peak force in each unfolding event was detected using an in-house written peak detection MATLAB script. The resulting forces were used in the force histograms and phase space analyses. To describe the dependence of the measured forces (*F*) on extension (*x*) for each peak, the rising phase of each sawtooth in the force-extension profiles (corresponding to the entropic-elasticity of unfolding) was fitted to a wormlike chain (WLC) model using in-house MATLAB software following [30]:

$$F=\frac{k_{B}T}{p}\left(\frac{1}{4{\left(1-\frac{x}{L_{c}}\right)}^{2}}-\frac{1}{4}+\frac{x}{L_{c}}\right)$$ (1)

where *p* (fixed at 0.36 nm) and *L*_*c*_ denote persistence and contour lengths, respectively, and *k*_*B*_*T* is the thermal energy (= 4.1*pN*·*nm* = 4.1·10^−12^*J* at room temperature). Loading rates, *lr*, were calculated as the product of the pulling velocity, v*, and the slope of the force-distance curve obtained from the best fit of Eq 1 to a given force peak [31]:

$$lr=v^{*}{\frac{dF}{dx}|}_{F=\langle F\rangle }=v*\frac{k_{B}T}{p}{\left(\frac{1}{2L_{c}}{\left(1-\frac{x}{L_{c}}\right)}^{-3}+\frac{1}{L_{c}}\right)}_{x=\langle x\rangle }$$ (2)

The difference between the contour lengths obtained for sequential peaks is the *ΔLc* used in the analysis. The most probable unfolding force, *F*_*p*_, and the most probable change in contour length *(ΔL*_*C*_) were determined by fitting the histograms of unfolding force and *ΔL*_*C*_ with two Gaussian functions. t-test of the Gaussian fitting was done with p (alpha)-values less than 1x10^-4^.

For the phase space construction we plotted all the measured pairs of unfolding force, *F*, and *ΔL*_*C*_ (Fig 1C panel I) and binned them using a bin size of 10 pN×5 nm. The bin-width of the force distributions (10 pN) was chosen as the root-mean square of the fluctuation of the unfolding force due to cantilever thermal fluctuations given by $\Delta F_{rms}\approx \sqrt{k_{c}\times k_{B}T}$. The bin width for the *ΔL*_*C*_ histograms was chosen as 5nm which is the smallest feature we could measure as determined using spherical beads. The binned data were plotted as contour plots, where the colour corresponds to the frequency of events in each bin (Fig 1C panel II). In addition to the contour plots, we constructed histograms of *ΔL*_*C*_ and *F* (panels I and II of Fig 1D respectively).

### Molecular dynamics: Pulling simulations

A coarse-grained model based on the native structure (Go-like potential) was used to simulate the mechanical unfolding of IFNAR1 with and without ligand [32]. We used a reduced representation of the protein in which each amino is represented by a single bead, centered at the Cα atom. The distance between successive Cα beads, σ, is 3.6 Å. The masses, *m*, of all the beads are identical. All secondary and tertiary native contacts between amino acids are represented by the Lennard-Jones potential without any discrimination between the various chemical types of the interactions. The energetic strength of all the contacts is thus identical and equal to ε. Additional details of the Hamiltonian of the system and its parameters can be found elsewhere [32, 33]. Brownian motion was added to the protein using the Langevin equation of motion. The system was propagated with time step τ. One may estimate the time unit in the simulation using the following relation τ = σ*m*^0.5^ε^-0.5^. Assuming the average mass of an amino acid to be 4.6x10^-26^ kg, and that ε = k_B_T = 0.6 kcal mol^-1^ (2510.4 J/mol), we find τ ~1 ps. This time unit describes the time scales of the CG beads, however, it underestimates the timescales for real proteins because the CG model has a reduced number of degrees of freedom and consequently a smoother protein energy landscape.

To allow pulling, the protein was connected to two harmonic springs; one at its C-terminal, whose end was kept fixed and, another, used for pulling (the pulling spring), was attached at several chosen positions [34–36]. Both springs were connected to the protein when it was in its native structure and pulling was simulated by moving the pulling spring along the axis connecting the springs' positions in the native state at a constant velocity of v_p_ = 5·10^−3^ Å/τ. During a simulation we monitored the instantaneous pulling force, *F*, which is the extension of the pulling spring times its force constant Kp (= 1 ε/Å^2^) as well as the extension of each domain which is the geometrical distance between the first and last residue of each domain.

To simulate the unfolding of IFNAR1-EC on its own we used the PDB structure 3S98 which contains the truncated form of IFNAR1-EC including only the three N-terminal domains (SD1-3). As a model of the bound state of the receptor we used the PDB structure 3SE3 which is taken from a crystal of the ternary complex IFNAR1-EC-YNS-IFNAR2, where we kept the coordinates only of IFNAR1-EC (three N-terminal domains).

## Results and discussion

For the experiments, the C-terminal end of the extracellular domain of IFNAR1 (IFNAR1-EC) was site-specifically attached to a mica surface through a flexible polymer linker (smcc-PEG7, Fig 1A). An AFM cantilever tip was brought into contact with the surface, where it became attached to the protein. As the protein is pulled the domains unfold, giving rise to force-extension peaks, which together produce a characteristic sawtooth pattern [37, 38]. In our experiments the interaction between tip and protein is non-specific, therefore the tip can attach to any of the domains of the protein and the force-extension curve can contain data from the pulling of one to four domains. Fig 1B shows traces representative of the different force-extension curves obtained at a pulling speed of 200 nm/sec. They exhibit the characteristic sawtooth pattern, but vary in the number of peaks observed and in the distances between peaks, indicating that they contain data from a varying number of domains and from unfolding through intermediates. Approximately 40% of the traces contained equidistant peaks varying in number from two to four (panel I of Fig 1B). The remaining curves displayed additional peaks, whose distance from other peaks was sometimes smaller than a single domain’s length. Of the different types of unfolding curves obtained, the most prominent form is that displayed in panel III of Fig 1B.

All unfolding forces measured, *F*, were in the range of 50–200 pN, consistent with unfolding of FNIII domains at similar pulling speeds [39–41]. The changes in contour lengths, *ΔL*_*C*,_ per peak that we measured were in the range of 5–50 nm. As a single domain of IFNAR1 spans approximately 35 nm, the *ΔL*_*C*_s we measured consist of a mixed population of partial and full unfolding of domains. The total length of the unfolded proteins did not usually exceed 120 nm, which is below the expected length of 150 nm for fully unfolded IFNAR1. This is mostly due to the presence of a disulfide bond within each of the FNIII domains that in total span a length of about 20 nm and cannot be pulled apart under the conditions of these experiments.

The phase space for unfolding (Fig 1C) was mapped by constructing a scatter plot of all the measured pairs of *F* and *ΔL*_*C*_ (panel I) and binning the data using a bin size of 10 pN×5 nm. The binned data were plotted as contour plots, where the colour corresponds to the frequency of events in each bin (panel II). In addition to the contour plots, we constructed histograms of *ΔL*_*C*_ and *F* (panels I and II of Fig 1D respectively).

**Fig 2:**
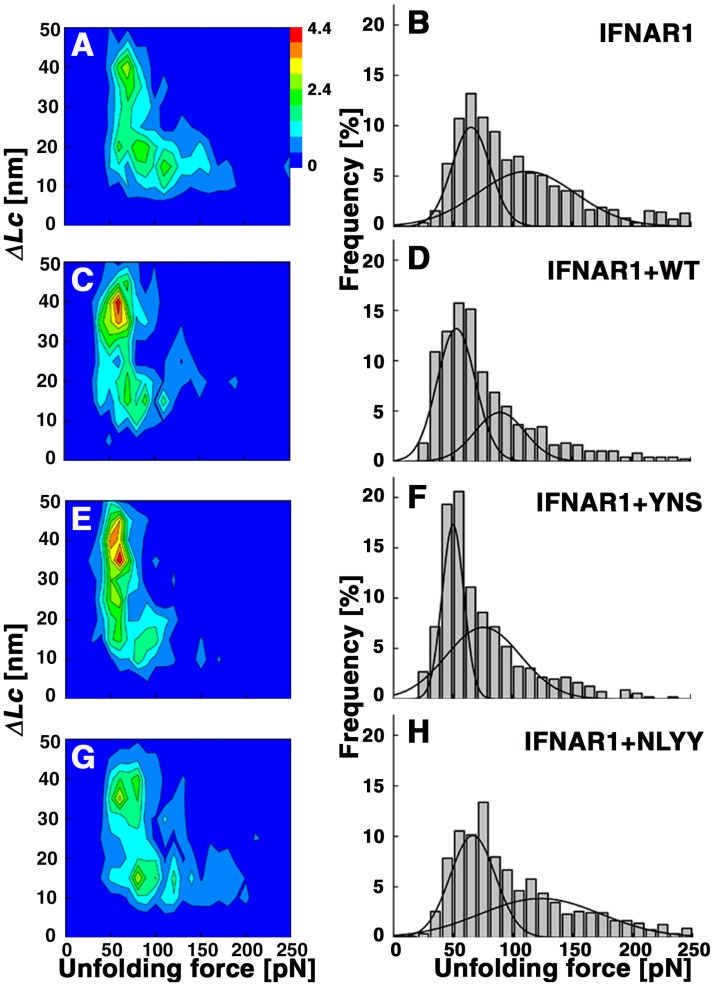
Influence of IFNα2 binding on IFNAR1 unfolding. Unfolding of IFNAR1-EC on its own (A and B,) or in the presence of 4 μM WT- IFNα2 (K_D_ = 1.5 μM, C and D), the high affinity mutant YNS (K_D_ = 0.03 μM, E and F) and the low affinity mutant NLYY (no measureable binding affinity [46], G and H). The influence of the ligands is described either by contour plots (C,E,G) or force histograms (D,F,H) each representing 3–4 experiments.

Two populations can be distinguished in each of the two dimensional phase plots presented in (Fig 2A, 2C, 2E and 2G) based on their differences in *ΔL*_*c*_. We therefore fit the ΔL_c_ data to two Gaussians (p-value < 10^−4^): One population is widely distributed and is centred on an increase in contour length of ~30 nm, the approximate length of a single domain of IFNAR1-EC, and the second population is centred at a lower value of ~16 nm and is distributed more narrowly. Looking at the contour plot (Fig 1C, panel II), we can identify that the forces that correspond to opening up full domains are ~50–100 pN, but higher forces are sometimes required to unfold the shorter features and these are distributed along a wider range. Consequently the histogram of unfolding forces was also fitted by two Gaussians (p-value < 10^−4,^ see Materials and methods).

We repeated the experiments in the presence of three different IFNs: Wild-type IFN α2 (*K*_*D*_: 1.5 μM), a triple mutant that binds to IFNAR1 50-fold stronger than the wild type called YNS [8], and a mutant whose binding affinity is too low to be measured (IFN-NLYY)[42], which served as a negative control.

Addition of wild-type IFNα2, surprisingly, reduced the forces required to unfold IFNAR1-EC and also reduced their range (Fig 2C and 2D). This effect is even more pronounced with the binding of the high affinity mutant, YNS (Fig 2E and 2F), and disappears with the addition of the low binding affinity mutant (Fig 2G and 2H). The decrease in unfolding forces is stronger for the high force peak in the histogram and there seems to be a shift of events toward low force/high *ΔL*_*C*_ events. The same behavior was observed at other pulling speeds (see S3 Fig). The dependence of the shift in the forces on IFN binding confirms that the effects observed are indeed the result of ligand binding. Previous forced unfolding of multi-domain proteins, with and without their ligand, showed either an increase in the force of unfolding [43] or no change [44, 45]. *To the best of our knowledge*, *this is the first experimental demonstration that ligand binding lends a protein more easily mechanically unfolded*. This effect may be linked to the surprising observation that the dissociation kinetics of the IFNα2-IFNAR1 complex decreases with increasing temperature resulting in a negative activation energy.

**Fig 3:**
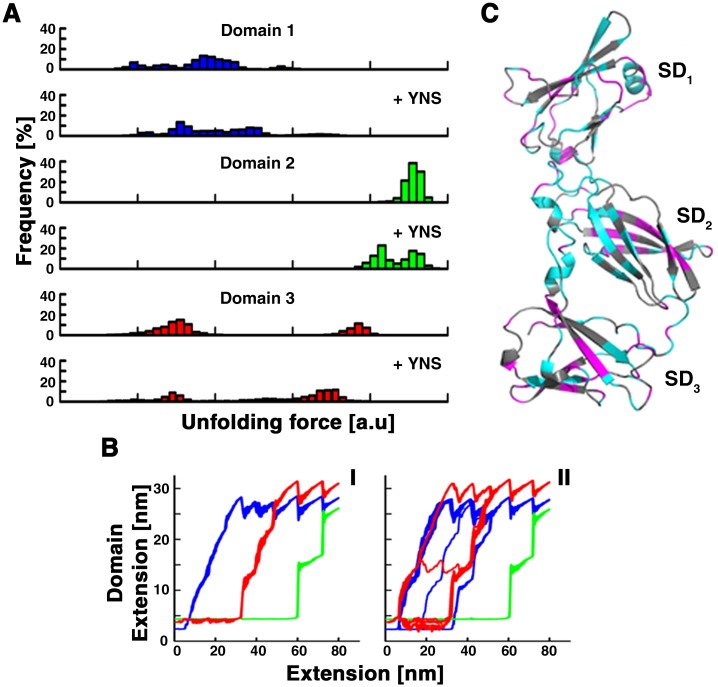
Coarse-grained MD simulations of IFNAR1-EC and contact changes affected by YNS binding. (A) Histograms of unfolding forces by domains (SD1 –blue, SD2 –green, SD3 –red), with the top graph representing unfolding of the domain on its own and the bottom graph showing the unfolding of the domain in the presence of YNS. (B). Traces of steered MD of IFNAR1-EC domains on its own (I) or with its ligand (II). I and II each consist of 60 traces with colours as in A. C. Residues that have lost contacts with other residues upon interaction with YNS are coloured in cyan whereas those that have gained contacts are coloured in magenta.

To better understand how binding of the ligand renders the receptor *less* resistant to force, we performed steered MD simulations [34–36] in which IFNAR1 in its unbound and in its YNS-bound form were unfolded mechanically. Simulation were performed using a coarse grained model based on the native topology [47] of the protein, either on its own or bound to YNS (IFNAR1-EC, ΔSD4: 3S98, Ternary complex: 3SE3). We note that both structures contain data for domains 1–3 only (SD1-SD3). In the simulations we pulled IFNAR1 through a spring attached to the protein, at various points, and monitored the magnitude of the force that was applied on the spring, the length of the protein chain and the end-to-end length of each domain (Fig 3B).

The data obtained from the simulations were analyzed in a manner similar to the experimental data. For the simulations we could identify which domain gave rise to each peak and thus were able to analyze the unfolding of each domain separately. Force curves were constructed using the force and domain extension data (Fig 3B and S2 Fig) and the same algorithm used to analyze the experimental data was applied to detect force peaks. The histograms of forces that unfold each domain are presented in Fig 3A. Within each panel we show the unfolding-force histogram of an IFNAR1 domain on its own on top and that of the same domain unfolding in the presence of YNS on the bottom. Fig 3BI shows the length of each domain *vs* the entire length of IFNAR1-EC throughout the pulling process. For IFNAR1 on its own, the order and path of unfolding of the domains was identical in all 60 simulations. The first to unfold was always SD1 (blue) in a multi-step process (see S2 Fig) that gave rise to a wide distribution of low forces in the histogram (Fig 3A, top blue). Next, SD3 unfolded in a sequence of three large steps (Fig 3BI, red) that lead to two, well separated peaks in the force histogram (Fig 3A, top red). Finally, SD2 unfolded in two large, similarly sized steps (Fig 3BI, green) that appear in the force histogram as one peak at high forces (Fig 3A, top green). In the presence of YNS, we observe some variation in the sequence of unfolding (Fig 3BII) where SD1 and SD3 may alternate but SD2 was always the last to unfold. The number of steps in the unfolding path of each domain was unchanged. A greater variability in step sizes could be observed in the unfolding path of SD1 as seen also in the histogram of unfolding forces (Fig 3A, bottom blue). The larger steps in the unfolding of SD3 occurred at slightly lower forces leading to a shift of the high forces in the histogram (Fig 3A, bottom red). The unfolding of SD2 again occurred last, in two steps, however, one of these occurs at a lower force leading to a splitting of the force histogram into two (Fig 3A, bottom green). The net result of these changes is that the forces needed to unfold the form of IFNAR1 that is bound to IFN are lower than those required to unfold IFNAR1 on its own. Note that all the forces calculated in the simulations originate from events in which partial unfolding of the domains occurred, as reflected by the resulting change in domain extension, which is always smaller than the 36 nm expected for full unfolding of a domain. This is corroborated by following the unfolding throughout the simulation. Note also that the temporal resolution of the simulations is significantly higher than that of the experiment, revealing details in the force histograms that are not observed in the experiment. Thus the partial unfolding events observed in the simulations most likely correspond to the low *ΔL*_*C*_/high F peaks obtained in the histograms of the experimental data and not to the full dataset as presented in Fig 2.

Overall, the experimental data, together with the simulations indicate that unfolding of IFNAR1 can occur either through complete unfolding of domains, or through intermediates. The latter, occur at higher forces that, surprisingly, are reduced when the ligand binds to the receptor.

One possible explanation for this reduction is that the binding of IFN leads to a conformational change that disrupts contacts within and between the domains, leading to a different unfolding path. Support for this comes from bulk measurements that have shown that the enthalpy for binding of IFN is positive, indicating that bonds in the receptor are broken during the interaction [22]. We analyzed the contacts formed or disrupted in IFNAR1-EC when it binds YNS (Fig 3C) using CSU [48]. We found that interaction with IFN leads to both disruption and addition of contacts within all three N-terminal domains of IFNAR1-EC. The net changes in number of contacts per residue are summarized in Fig 3C, where residues that have overall gained contacts are coloured magenta and those that have lost contacts are coloured cyan. For SD1 and SD3 there seems to be no apparent spatial pattern either in the addition or reduction of net interactions. For SD2 on the other hand, almost all residues that have overall gained intra-protein contacts are located in one of the beta sheets whereas almost all those that have lost contacts are located in the other. Thus one of the beta sheets unfolds at forces that are lower than the ones that unfold the other, leading to the two, clearly distinguishable peaks in the force histogram (Fig 3A, green bottom) with the net result being an overall reduction in unfolding forces.

Another possible explanation for the reduction in unfolding forces for the IFN-bound structure is that binding of IFN disturbs interactions between domains of IFNAR1 that have been suggested to stabilize multi-domain proteins [49].

Altogether, Fig 3C suggests that the interface formed between IFNAR1 and IFN disturbs the network and number of the intra- and inter- domain interactions. This redistribution of the internal energy, induced by ligand binding, can change both the mechanism and force of unfolding.

## Conclusions

Recognition of IFNs by IFNAR1 has probably evolved to cover a large variety of binding affinities and stabilities, thus providing functional selectivity for fine-tuned cellular responses against different pathogens [14]. Structural studies suggested that functional selectivity may be encoded in complex conformational changes that propagate within IFNAR1-EC upon ligand binding. With the aim of better understanding the structural basis of these effects, we have explored changes in the mechanical stability of IFNAR1-EC upon ligand binding. Interestingly, we find that upon binding of IFN to IFNAR1, it is more easily unfolded by force. To our knowledge, ligand binding was hitherto shown to result in either stabilization [43] or unchanged stability [44, 45] of the cognate protein, which is expected as binding energy may need to be overcome. In case of the IFN-IFNAR complex, the loss in mechanical stability is probably related to the ligand-induced conformational reorganization within the four FNIII-like domains. The shift to lower forces occurs mainly through changes in SD2 and SD3 and to a lesser extent in SD1. The unusual destabilization of IFNAR1-EC by interaction with IFN is in line with the likewise unusual negative activation energy observed in temperature-dependent ligand binding assays [22]. Our studies thus further highlight the unique nature of the IFN-IFNAR1 interaction, which is weak but involves large-scale conformational rearrangements. Since inter-domain movement was suggested to be responsible for propagating the signal from the extracellular to the intracellular domains of IFNAR1, it is possible that the stiffer conformation of IFNAR1-EC on its own prevents it from undergoing unwanted thermal conformational changes that would accidentally trigger the downstream signaling cascade. Binding of IFN “softens” the protein facilitating transduction of the signal. This mechanism is in line with a recently proposed inactive conformation of the functionally related epidermal growth factor receptor, highlighting a key role of conformational changes in transmembrane signaling [50]. IFNAR is a paradigm of functional selectivity of cytokine receptors, yet highly similar properties are currently emerging for several other cytokines such as IL-2/IL-15 [51], IL-10 [52, 53], IL-4 [54, 55] and erythropoietin [56] that further corroborate the important role of receptor recognition dynamics for functional selectivity.

## Supporting information

S1 Fig Activity of purified IFNAR1-EC as measured by column gel filtration. Retention pattern of high affinity interferon YNS when injected along with IFNAR1-EC into a Sepharose Gel Filtration column. Y axis, detection of protein flow at 280nm. The complex with YNS is located as reported previously [8].
(TIF)

S2 Fig Force-extension curves obtained in the MD simulations. Panels A and B show extension curves and panels B and D their respective force curves, from which we obtained the most probable force for unfolding for each domain.
(TIF)

S3 FigForce spectra for low (A) and (B) high force peaks of IFNAR1-EC on its own or in the presence of IFN (WT, YNS and NLYY).IFNAR1-EC was pulled at different pulling speeds, ranging from 100 to 10000 nm/sec with a cycle amplitude of 250nm. Most probable force for unfolding was obtained as described in the “Materials and Methods” for pulling at 200 nm/sec. The dependence of the most probable force, *F*_*p*_, on the loading rate was fitted with the Bell-Evans model [57] described by: $F_{p}=\frac{k_{B}T}{x_{u}}\text{ln}\left(\frac{x_{u}lr_{p}}{k_{B}Tk_{u}}\right)$, where *x*_*u*_ is the distance from the free energy minimum to the transition-state barrier (unfolding barrier) along the reaction coordinate, *k*_*u*,_ the rate of unfolding in the absence of applied force, and *lr*_*p*,_ the loading rate. The force of unfolding for IFNAR1 on its own is higher than that of IFNAR1 bound to WT-IFN, and YNS and similar to the low affinity mutant NLYY. This results holds for both force peaks and at all pulling speeds.(TIF)
