# Supplementary figures and images for: Binding of interferon reduces the force of unfolding for interferon receptor 1
Source: PLoS One. 2017 Apr 12;12(4):e0175413. doi: 10.1371/journal.pone.0175413 (PMC5389645; doi:10.1371/journal.pone.0175413)

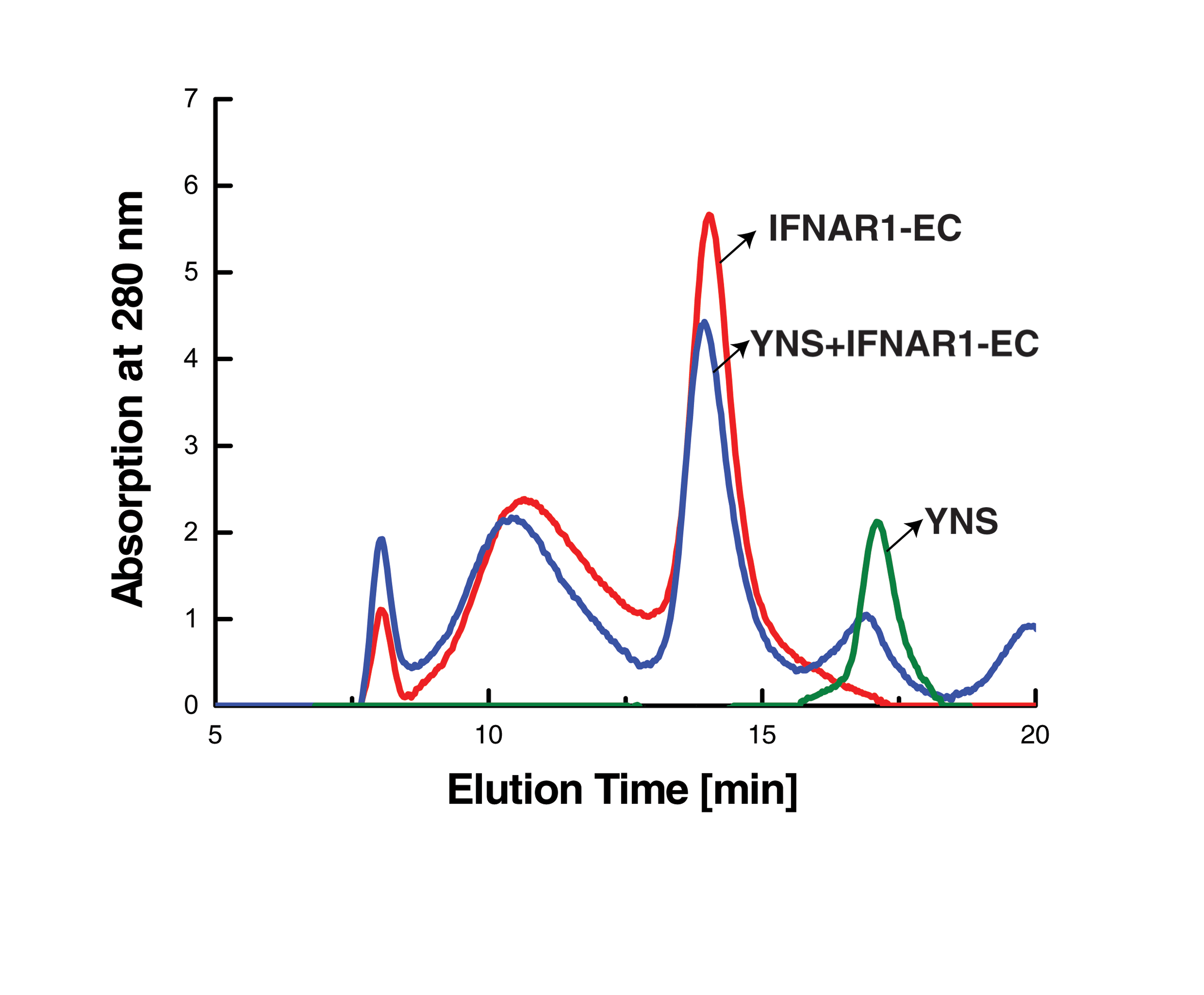

Supplement: S1 Fig — Retention pattern of high affinity interferon YNS when injected along with IFNAR1-EC into a Sepharose Gel Filtration column. Y axis, detection of protein flow at 280nm. The complex with YNS is located as reported previously [8]. (TIF) [file pone.0175413.s001.tif]

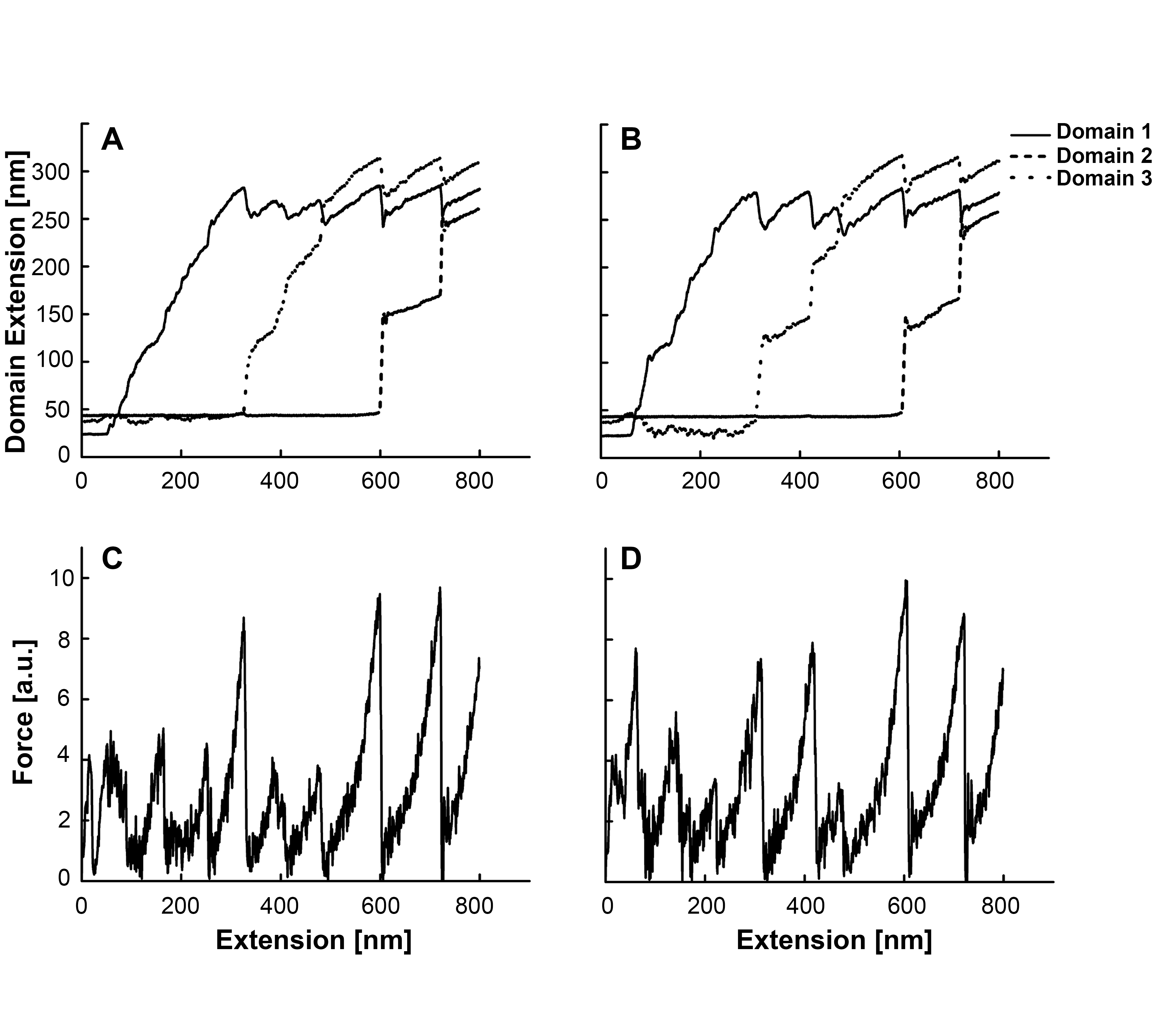

Supplement: S2 Fig — Panels A and B show extension curves and panels B and D their respective force curves, from which we obtained the most probable force for unfolding for each domain. (TIF) [file pone.0175413.s002.tif]

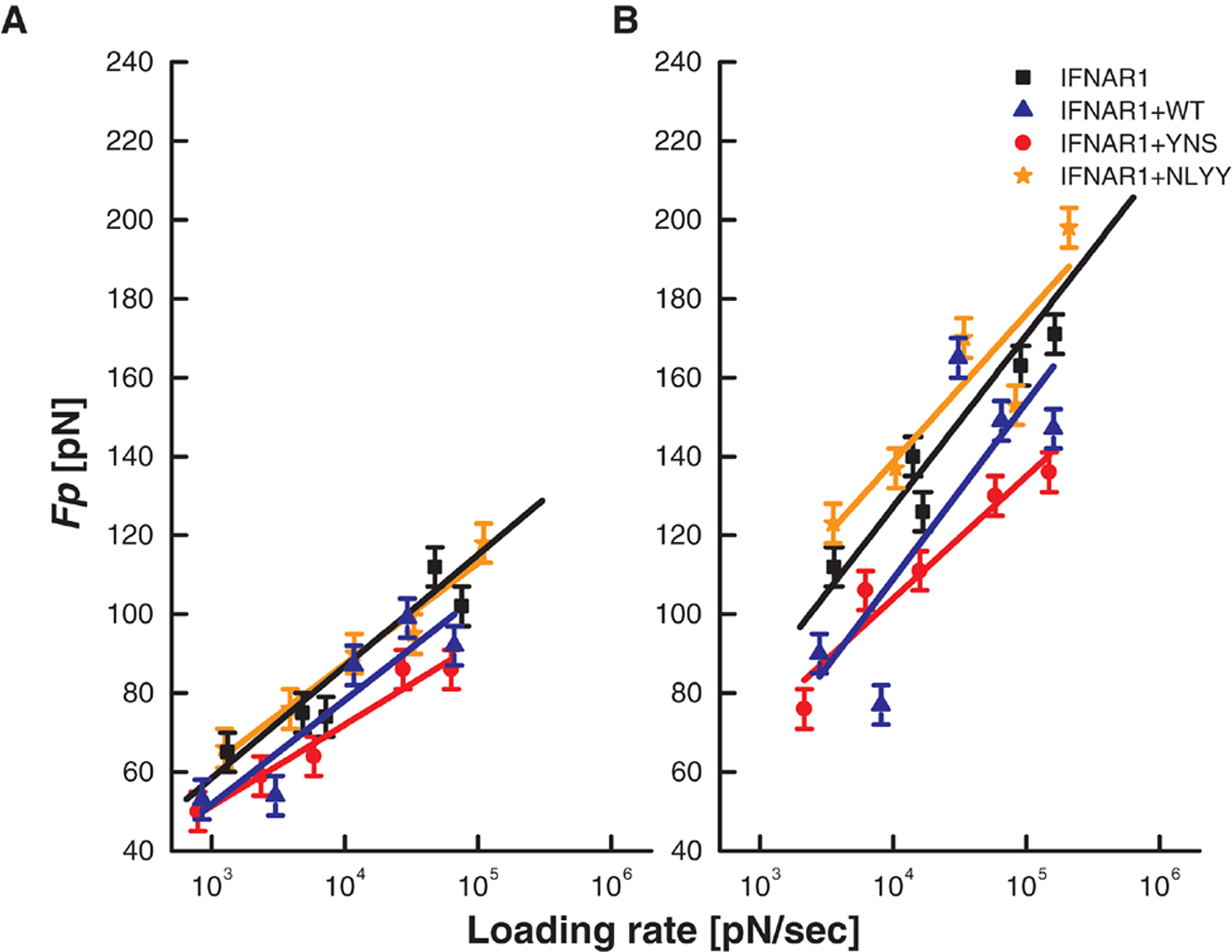

Supplement: S3 Fig — IFNAR1-EC was pulled at different pulling speeds, ranging from 100 to 10000 nm/sec with a cycle amplitude of 250nm. Most probable force for unfolding was obtained as described in the “Materials and Methods” for pulling at 200 nm/sec. The dependence of the most probable force, Fp, on the loading rate was fitted with the Bell-Evans model [57] described by: Fp=kBTxuln(xulrpkBTku), where xu is the distance from the free energy minimum to the transition-state barrier (unfolding barrier) along the reaction coordinate, ku, the rate of unfolding in the absence of applied force, and lrp, the loading rate. The force of unfolding for IFNAR1 on its own is higher than that of IFNAR1 bound to WT-IFN, and YNS and similar to the low affinity mutant NLYY. This results holds for both force peaks and at all pulling speeds. (TIF) [file pone.0175413.s003.tif]
